# A New Approach in the Design of Microstructured Ultralight Components to Achieve Maximum Functional Performance

**DOI:** 10.3390/ma14071588

**Published:** 2021-03-24

**Authors:** Amaia Calleja-Ochoa, Haizea Gonzalez-Barrio, Norberto López de Lacalle, Silvia Martínez, Joseba Albizuri, Aitzol Lamikiz

**Affiliations:** 1 Department of Mechanical Engineering, University of the Basque Country (UPV/EHU), Nieves Cano 12, 01006 Vitoria-Gasteiz, Spain; 2 Department of Mechanical Engineering, University of the Basque Country (UPV/EHU), Ingeniero Torres Quevedo Plaza, 1, 48013 Bilbao, Spain; haizea.gonzalez@ehu.eus (H.G.-B.); joseba.albizuri@ehu.eus (J.A.); aitzol.lamikiz@ehu.eus (A.L.); 3 Centro de Fabricación Avanzada Aeronáutica-CFAA, University of the Basque Country (UPV/EHU), Parque Tecnológico de Zamudio 202, 48170 Bilbao, Spain; norberto.lzlacalle@ehu.es (N.L.d.L.); silvia.martinez@ehu.eus (S.M.)

**Keywords:** inconel 718, additive manufacturing, powder bed fusion, microstructures, replication, heterogeneous geometries

## Abstract

In the energy and aeronautics industry, some components need to be very light but with high strength. For instance, turbine blades and structural components under rotational centrifugal forces, or internal supports, ask for low weight, and in general, all pieces in energy turbine devices will benefit from weight reductions. In space applications, a high ratio strength/weight is even more important. Light components imply new optimal design concepts, but to be able to be manufactured is the real key enable technology. Additive manufacturing can be an alternative, applying radical new approaches regarding part design and components’ internal structure. Here, a new approach is proposed using the replica of a small structure (cell) in two or three orders of magnitude. Laser Powder Bed Fusion (L-PBF) is one of the most well-known additive manufacturing methods of functional parts (and prototypes as well), for instance, starting from metal powders of heat-resistant alloys. The working conditions for such components demand high mechanical properties at high temperatures, Ni-Co superalloys are a choice. The work here presented proposes the use of “replicative” structures in different sizes and orders of magnitude, to manufacture parts with the minimum weight but achieving the required mechanical properties. Printing process parameters and mechanical performance are analyzed, along with several examples.

## 1. Introduction

In energy, aeronautical, aerospace industries and others, many opportunities for advanced materials research are currently open. The high-temperatures in energy components lead to use new materials with enhanced capabilities, especially nickel-based superalloys and titanium alloys. Superalloys are used for gas turbine blades, compressor blades, and heat shields, because they have very good mechanical properties up to 700 °C.

This work is focused on the nickel-based superalloy called Inconel 718 (Table 1). Introduced in 1965 on an industrial scale, alloy Inconel 718 is relatively widespread in the energy sector, especially for the manufacture of the so called “hot areas components”. Indeed, around 50% of produced Inconel 718 is used in engine (turbine) manufacturing, including blades, seals, and discs [1]. Moreover, Inconel 718 is commonly used by applying additive manufacturing technologies because of the alloy appropriate melting and solidifying properties.

The effect of process parameters on residual stresses, distortions, and porosity in Selective Laser Melting (SLM) was studied [2] for Maraging Steel 300, and in [3] for Inconel 718 (co-authors previous work). Moreover, some studies also worked on the effect of the ti6al4v alloy track trajectories on mechanical properties in direct metal deposition [4], the effect of microstructure, crystallographic texture and morphological texture on the mechanical properties of 3D printed 316L stainless steel [5] and the effect of scanning strategy on grain structure and crystallographic texture of Inconel 718 processed by selective laser melting [6]. Some researchers also worked on the effect of build orientation on the corrosion behavior and the mechanical properties of Selective Laser Melted Ti-6AI-4V [7]. The influence of heat treatments on heat affected zone cracking of gas tungsten arc welded additive manufactured alloy 718 was also investigated [8].

On the other hand, cellular lattice structures have interesting applications in energy, aerospace, automobile and defense industries due to their high “specific strength” (the ratio strength/weight), elastic modulus and energy absorption [8]. In addition, different sectors demand lighter materials and structures that will maintain component strength but reducing its weight and, this is the case of microstructures.

Microstructures are formed by rigid skeletons that are able to maintain the global component stiffness. The main challenge is to design with optimized microelements size, shape, and topology. The manufacturing of these microstructures formed by a heterogeneous skeleton with cavities is an actual challenge for additive manufacturing technologies. Precisely, the design of porous and lattice-like structures goes along with the blossoming of additive manufacturing technology over the last 20 years. In fact, this type of structure already exists [9,10,11], but component properties are not well controlled. The microelements can be minimal surfaces with a fixed topology [9], and only some heuristics rules are used to control the shape of the micro-element.

The European market has some limited use of microstructures too, typically in grid-like (or lattice-like) arrangements. In recent years, there has been an increasing interest in curved geometries modeling using microstructures and auxetic materials for the manufacturing of highly complex porous objects using additive manufacturing. Thus, stochastic methods [12], Voronoi tessellations [13] and implicit surfaces [14] are a few examples of the variety of approaches that were aimed at modeling such geometries. However, these schemes are complicated to analyze and control, not to, say, employ inside the full design cycle. Recently, ability was introduced to precisely synthesize freeform repetitive parametric microelements as complete microstructure of arbitrary freeform shapes [15,16]. The synthesized geometry can be heterogeneous [17] using a volumetric geometric modeling representation (V-rep) that was introduced in [18] and is fully compatible with contemporary boundary representation (B-rep) design and modern (iso-geometric) analysis tools.

Regarding components of stochastic porous material manufactured by SLM, the influence of laser parameters and scanning strategies on the mechanical properties is studied in [19,20] and anisotropy is tested in [21]. However, these studies based on stochastic porous components manufacturing do not focus on structured geometries or micro-geometries and are not printed in Inconel 718.

Therefore, the manufacturing process of these microstructures is still a complex procedure, associated with one particular type of microstructure, leading to just one specific manufacturing algorithm and technique.

In this work, a methodology for the design and manufacturing of microstructured ultralight components to achieve maximum functional performance is proposed. Firstly, design and manufacturing process parameters for Laser Powder Bed Fusion (L-PBF) technology are defined. Secondly, the components behavior regarding compressive loads, and stress and strain distribution performance is analyzed by finite element simulation and experimental validation.

This study will result in a geometric modeling framework that will allow the design, analysis, optimization, and manufacturing of highly complex porous and microstructured heterogeneous geometries.

## 2. Design of Replicative Structures

The proposed structures are very light ones, in which the ratio strength/weight is brought to extreme limits. The idea is based on replicable basic structures in different orders of magnitude. The manufacturability by Laser Powder Bed Fusion (L-PBF) is analyzed, achieving the basic parameters to get right parts: orientation, laser power, pulsing time, bar thickness and the number of layers.

**Figure 1:**
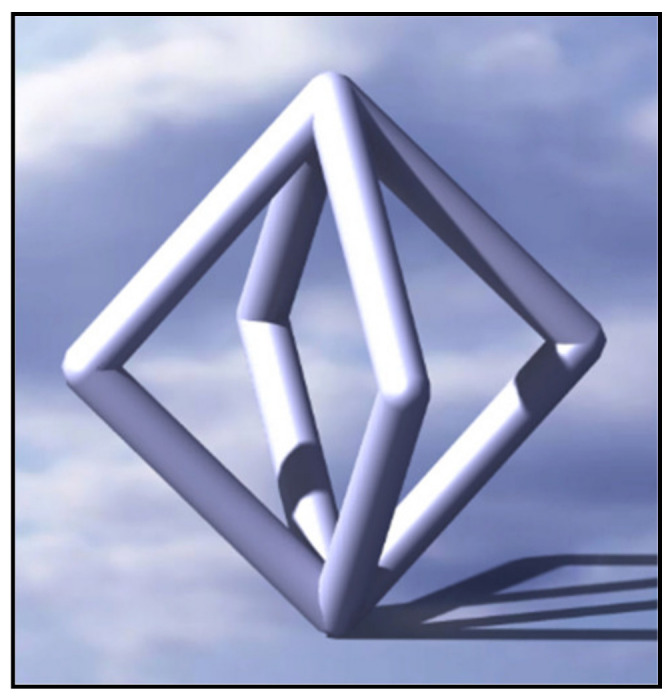
Octahedron at the first scale; the “cell”.

**Figure 2:**
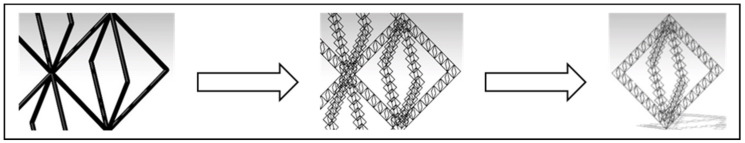
Three different scales of the structure. (**Left**) First Scale: small octahedron (“cell”), (**Center**) Second scale: octaedron lattices, (**Right**) Third scale: octahedron structure.

The final structure is a three-scale one: in our case, the general structure is the third scale, an octahedron made of octahedrons lattices (second scale), which are composed of even smaller octahedrons (“cells”) (Figure 1), which are the first scale, as the ones shown in Figure 2. The cell octahedrons are designed without horizontal bars due to L-PBF limitations to print this in the smallest scale.

Table 2 shows the parameters used for octahedrons design. Three parameters define the basic cell geometry of each structure: bars length, bars radius, and the number of octahedrons. The radius of the bar will depend on the process parameters since it is the radius of the melted area at a point. Higher power values and longer times will provide larger radius values. In the CAD very small radius values are designed on purpose so that the trajectory generation program can set an only point per bar and layer.

## 3. L-PBF Process Parameters

The pieces are printed in a Renishaw A400 machine with inert gas (argon) atmosphere. They are printed in Inconel 718 powder, layer by layer. Regarding powder information, powder parameters to define powder granulometry are listed below:

- Laser size diffraction test—ASTM B822:
  - Dv (10) = 26 µm; 10% of powder volume has a diameter of less than 26 µm.
  - Dv (50) = 37 µm; the center of the volume distribution in volume is 37 µm.
  - There is no dust below 15 µm in volume.
- Sieve analysis test—ASTM B214:
  - No dust above 45 µm by weight.

In order to obtain workable prints, different process parameters (Table 3) were tested. The printing strategy followed is the “SINGLE POINT STRATEGY” that corresponds to the “BLOCKED PATH” strategy that is used for very thin walls, in which only one line per layer and wall is manufactured, the other direction has been restricted and, then, only one point per layer and bar is manufactured. Layer thickness is 30 microns. For the “SINGLE POINT STRATEGY” the only parameters that need to be programmed are laser power and laser exposure time at each point (Table 3).

**Figure 3:**
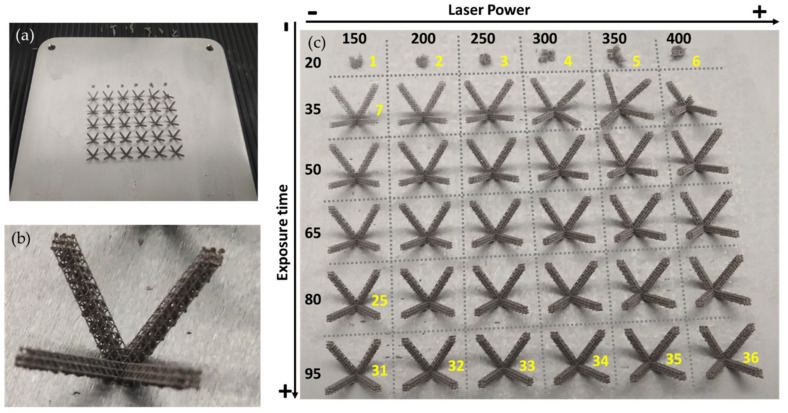
(**a**) Printed geometries for parameter tests. (**b**) Zoom on printed geometry for parameter tests. (**c**) Printed geometries organized according to tested parameters.

The first step was to find optimized parameters of the L-PBF process to print octahedron structures. Printings were performed for specific values of laser power and exposure time. As shown in Table 3, the laser power range is between 150 and 400 W, the exposure time range is between 20 and 95 µs. The first prints were carried out for each combination of parameters (Figure 3).

**Figure 4:**
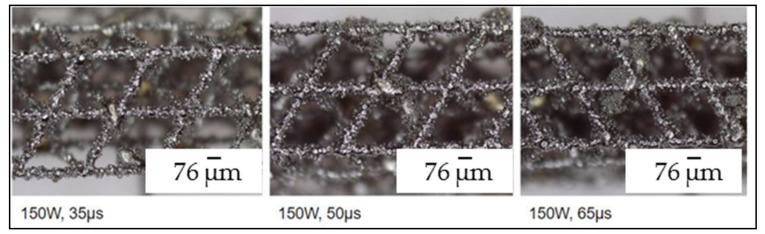
Printed workpieces. (**Left**) Microstructure printed with 150 W laser power and 35 µs of exposure time. (**Center**) Microstructure printed with 150 W laser power and 50 µs of exposure time. (**Right**) Microstructure printed with 150 W laser power and 65 µs of exposure time.

The best parameters (according to printed workpieces results) were 150 W of power (the lowest tested) and 55 μs exposure as shown in Figure 3. Finally, optimized process parameters are shown in Table 4. In Figure 4, printed microstructures with different settings can be seen in microscope (Alicona 5U, Raaba, Austria) images.

**Figure 5:**
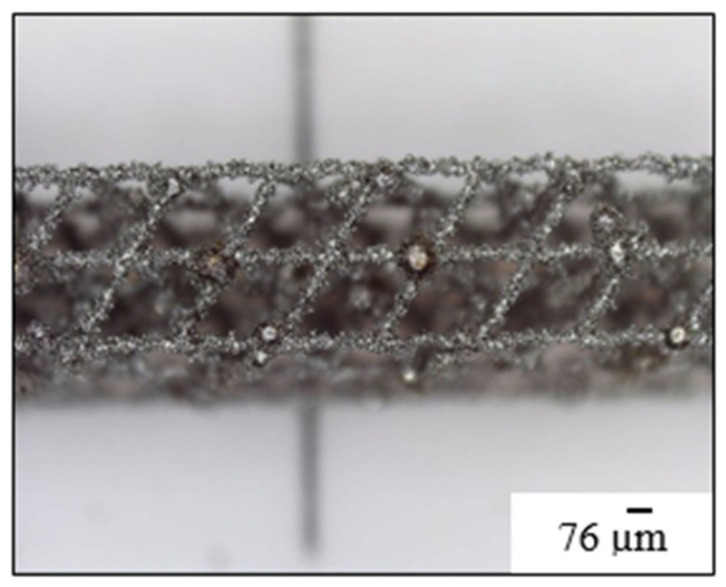
Microscope image of octahedron structure with metal spheres.

Regarding workpiece geometry, after image microscopy analysis, authors noticed that the horizontal bars in the smallest scale presented many printing problems. The “balls” in Figure 5 are a consequence of design error. The bars were not made sufficiently fine and in the connection, as the area is larger, the program did not recognize it as a blocked path and used a normal strategy by sweeping areas and with contours. Joining areas were overheated.

**Figure 6:**
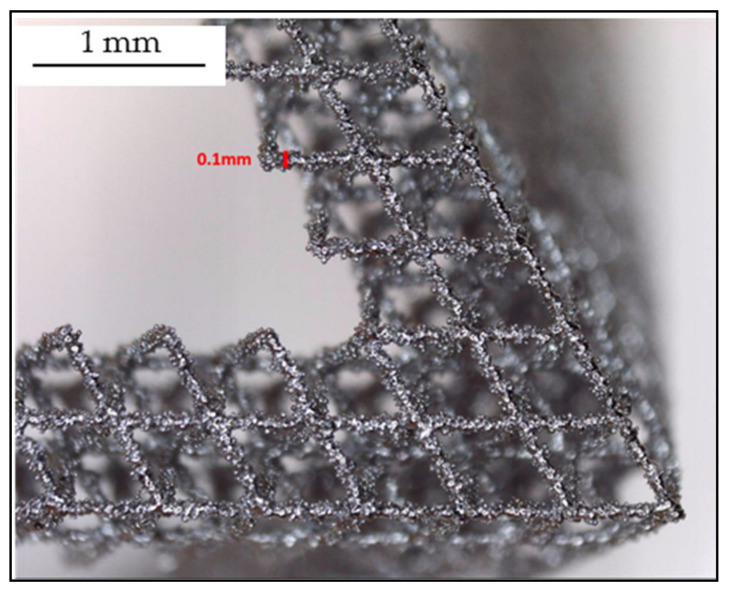
Microscope image of measured bars.

On the other hand, due to L-PBF process characteristics, powder was adhered (heat and affected zone) to the bars, and, measured resulting diameter (Figure 6) was 0.1 mm (5 times bigger than programmed one). This consideration is taken into account in Section 4.3 “Design of complex pieces: ‘fitting factor’ factor”.

## 4. Microstructures Performance Results

**Figure 7:**
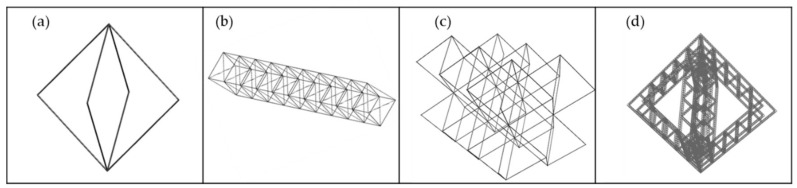
(**a**) Single octahedron design, (**b**) Octahedron microstructure bar made of 9 octahedrons, (**c**) 6 octahedrons microstructure, (**d**) Big octahedron microstructure made of octahedron bars and octahedron cells.

This section includes microstructures performance analysis: first, FEM (finite element method) simulation (Finite Element) under compressive load (Section 4.1), and, then, under experimental compressive load. Regarding Section 4.1, in order to analyze microstructures performance (compressive behavior) four different cases (Figure 7), from the simplest one to the most complex one, are designed. The first design (a) is a single octahedron, the second design (b) is a bar made of nine single octahedrons, the third design (c) corresponds to a six-octahedron microstructure, and the last design (d) is an octahedron made of bars (bars using the second design).

**Figure 8:**
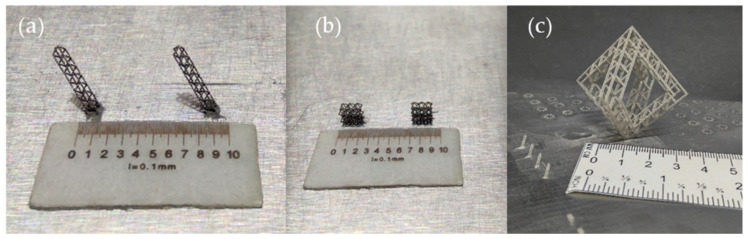
(**a**) Octahedron microstructure latice made of nine octahedrons, (**b**) 6 octahedrons microstructure, (**c**) octahedron microstructure made of octahedron lattice.

In Figure 8, real geometries after L-PBF manufacturing process can be seen. As it can be seen in Table 5, the dimensions of geometries a, b and c are geometries of relatively small size in comparison to geometry d. Therefore, in Section 4.2, where experimental validation of microstructures performance is presented, only geometry d is tested. In fact, geometries a and b are simple microstructures that will form part of geometry d.

**Figure 9:**
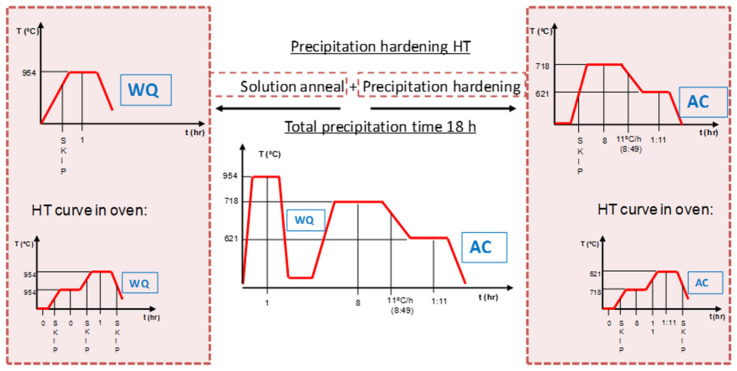
Two heat treatments suitable for IN718 [22].

On the other hand, in order to obtain Inconel 718 hardness values, manufactured components are subjected to a tempering treatment [22]. As it can be seen in Figure 9, (the abscissa axis is not absolute, it is relative), the component is introduced in the oven at 954 °C and solubilized for 1 h water quenching (WQ). Then, precipitate 8 h at 718 °C, reduce at 11 °C/h to 621 °C, which lasts 8:49 h, and maintain those 621 °C for a total time of 18 h, that is, 1:11 h remaining. Then, the component is air cooled (AC).

### 4.1. Performance under Compressive Loads

Finite element simulation (FEM) was carried out with ANSYS WB (Workbench) software. Simulated material is Inconel 718. A compressive load of value 1 N was applied between two opposite diagonal corners, and displacement restriction was applied in one corner. Regarding the geometry, structures were simplified to beam type elements instead of volume elements, for a faster analysis. The beam element diameter was set to 0.1 mm. In this case, this value is bigger than initial design but it matches with bars measured diameter after microstructure manufacturing process.

**Figure 10:**
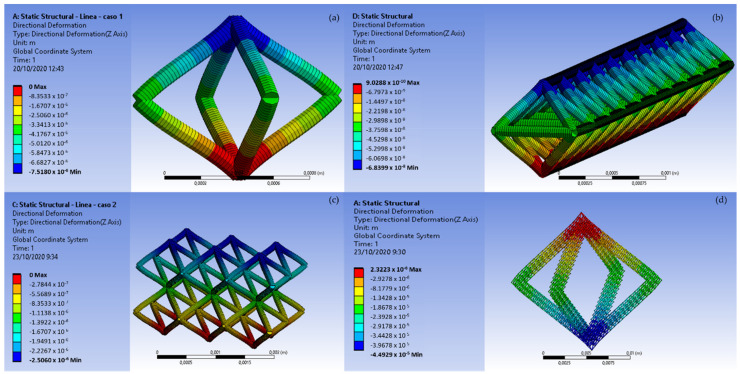
Directional deformation (*Z* axis), of the four designs (**a**–**d**).

Table 5 shows geometry properties regarding volume, mass, directional deformation in z-axis and minimum and maximum combined stresses (with resistance stresses, those due to the composition of axial stress and bending moment, there is no torsion and the shear stress is zero in the lower end). As shown in Figure 10, geometry d is the one with better performance regarding to directional deformation. However, geometry c presents almost similar values.

Regarding the “specific modulus”, this is a materials property consisting of the elastic modulus per mass density of a material. It is also known as the stiffness to weight ratio or specific stiffness. The concept in our case must be translated into two concepts, because density is not a proper concept in a near-to-empty structures (a to d), thus the following ones are more representative:

- Stiffness/Weight = (F/δ)/W. This can be calculated from FEM and experimentally, considering elastic behavior of the structures.
- Maximum load before collapsing/weight: Fmax/W. This was calculated experimentally, being the final criteria when structures are on the verge of being crushed under load.

As it can be seen in Table 5, the relation load/structure weight (kg/kg) is around 124 for a third order microstrure (geometry d). The same relation, for a solid geometry (with a volume of 19 × 104 mm^3^, 15.6 g mass and suffering 2 × 10^−2^ mm of directional deformation in the *z* axis), is around 6.54 × 10^−3^. The relation Young Module/structure weight (Gpa/kg), is around 2.56 × 10^5^ for the microstructure, and is 13.5 for the solid geometry. So, considering geometry and material analysis in the ratios load/weight and Young Module/weight, it can be said that studied microstructure presents 20,000 times better behavior that the solid microstructure.

### 4.2. Experimental Performance

Experimental procedure was carried out with different loads, in order to validate microstures’ behavior simulations. In this case geometry d was the one selected for experimental validation taking into account that geometries a, b and c are very small.

**Figure 11:**
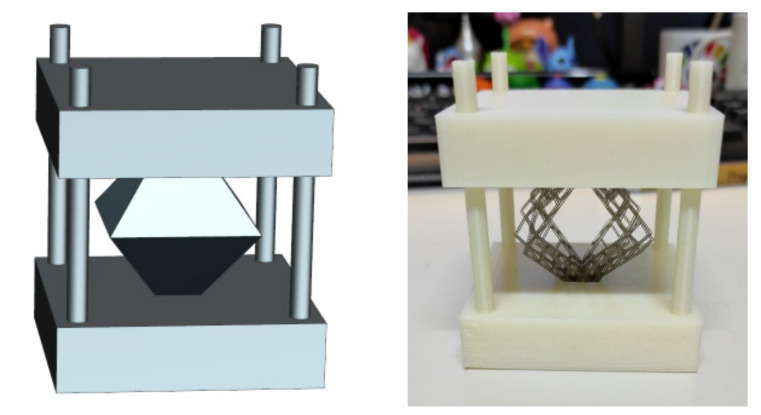
(**Left**) Designed structure for experimental validation. (**Right**) Real structure for experimental validation.

Regarding the experimental set up, designed structure (Figure 11) restricts “x” and “y” axes movement, as well as the rotary movement, to replicate the FEM simulation.

The octahedron is embedded in the upper and lower structure plates. The structure also presents four cylinders to guide the upper plate when the load is applied.

**Figure 12:**
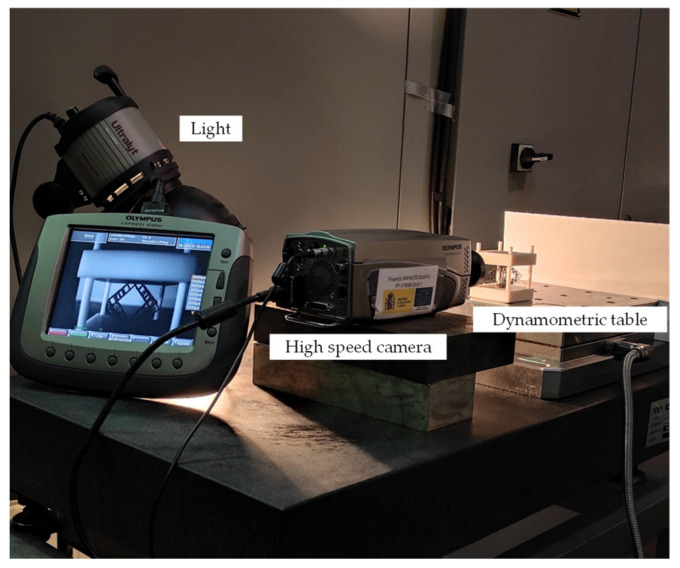
Experimental measuring set-up.

On the other hand, regarding the displacement measuring system (Figure 12), the experimental process is measured by a high speed camera. For each tested load, three different images are taken: one before the load, one with the load (these two will provide the displacement value) and one after the load withdrawal (in order to see whether there is elastic behavior or not). In order to take the different distances values, a function for measuring distances in images has been programmed in the Matlab software, with which the image has been processed. Initially, the measuring function is validated for two calibrated metrology gauges. Afterwards, for each image, three measurements of a known measure, such as the upper plate, are taken in order to calibrate and align the image. Then, the mean value for the distance between the two plates is taken three times.

**Figure 13:**
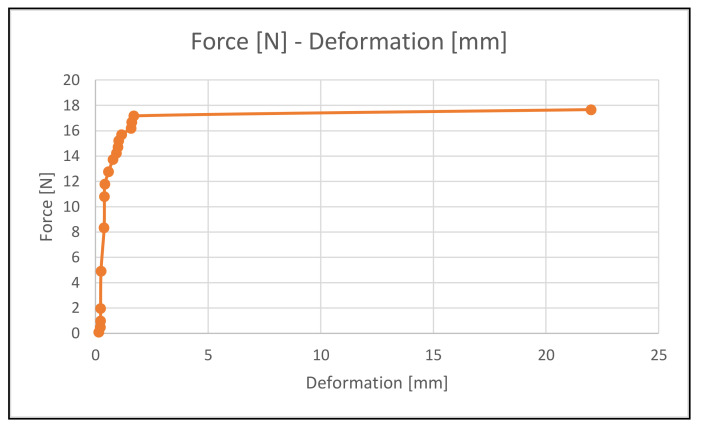
Microstructure Force-Deformation curve.

For the experimental test, different progressive loads are tested and each corresponding deformation is measured. Specifically, 18 loads (from 0.1 kg to 1.8 kg) are tested. As can be seen in Figure 13, the displacements increase from 0.1 to 1.9 mm up to 1.75 kg. Up to this point the microstructure presents elastic behavior. Then, for 1.8 kg, the microstructure fails and the displacement shot up to 22 mm.

### 4.3. Design of Complex Pieces: “Fitting Factor” Factor

In order to extrapolate the previous results to structures of different size and shape, it is necessary to establish a relationship between a) the finite element model and b) experimental validation of the simple structure, using a so-called “fitting factor”. This factor considers the difference between the designed part and the manufactured part due to the intrinsic characteristics of the L-PBF manufacturing process. Diameter affects stiffness in the third order, being a very sensitive parameter. The easiest way to do is to work using the bars diameter. Thus, using experimental and FEM tests, the real structure stiffness can be matched if a correction on bar diameter is considered. Fitting factor K-bar diameter = (9.90–10.10) variations are due to different lectures of deformation in the experimental tests.

Other possibility would be to consider both bar diameter on one hand, and right angle flexibility of joint connections of bars on the other, left for further research and discussion. However, right angle joints are in appearance stiffer than bars.

**Figure 14:**
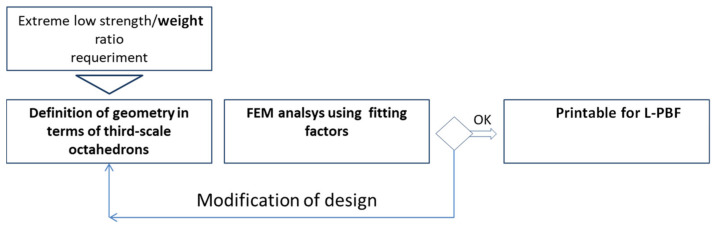
Proposed method for design using third-scale octahedrons.

With this “fitting factor”, parts that are more complex could be designed, and printed; the method is showed in Figure 14.

## 5. Discussion

Replicative structures are a very challenging application to achieve extreme ratios of strength/weight. Design method can be generalized to many applications, and it will be the object of further work inside the FET ADAM^^2^ (H2020-FETOPEN-2018-2019-2020-01 ADAM2 PROJECT) project.

In this work, microstructures design according to L-PBF technology printing limits are explored. In this sense, horizontal bars in the smallest scale presented many printing problems. There were also “balls” adhered to the bars as a consequence of design error. The bars were not made sufficiently fine, and, in the connection, as the area is larger, the program did not recognize it as a blocked path and used a normal strategy by sweeping areas and with contours. Joining areas were overheated.

Regarding geometry and material analysis in the ratios load/weight and Young Module/weight (Section 4.1), it can be said that studied microstructure presents 20,000 times better behavior that the solid microstructure. Indeed, the maximum load before collapsing is limited.

FEM can be a good choice for stress behavior simulation, being bars diameter the parameter to be adjusted by means of experimental validation. Once adapted for a stereotype-basic structure (such as the above explained cell and second-scale bar), it could be used in whatever piece design. Therefore, one octahedron bar of the second scale can be considered as the basic structural elements of any new piece to design. In the studied case, the third-scale geometry was an octahedron, but other substructures can also be used.

## 6. Conclusions

In this work, the idea of reducing weight in high strength elements using replicative structures is discussed, using P-LBF as basic manufacturing technology. The work main novelty is concluded hereafter:

- A methodology for the design and manufacturing of micro-structured ultralight components to achieve maximum functional performance is stablished.
- Design considerations for microstructures are stated. Component design is based on making replicas of the same structures, using octahedrons cells, from the smallest to the biggest scales.
- Manufacturing process parameters for microstructured ultralight components using Laser Powder Bed Fusion (L-PBF) technology are defined. Specific data for the cases studied are presented, and the boundaries in the print constraints are shown.
- Component behavior regarding compressive loads, and stress and strain distribution performance is analyzed by finite element simulation and experimental validation. Considering geometry and material analysis in the ratios load/weight and Young Module/weight (Section 4.1), it can be said that studied microstructure presents 20,000 times better behavior that the solid microstructure.
- A “fitting factor” in order to consider the difference between the designed part and the manufactured part due to the intrinsic characteristics of the L-PBF manufacturing process is considered. This factor is based on the bar diameter that affects stiffness in the third order, which is a very sensitive parameter. This method presents the correlation of FEM to real printing structures, which is easily done by a factor affecting bar diameter.

## Figures and Tables

**Table 1:** Inconel 718 chemical composition (wt.%).

Ni	Cr	Fe	Cb	Mb	Co	Al	Ti	Si	Mn	C
52.82	19.0	17.0	5.0	3.0	1.0	0.8	0.6	0.35	0.35	0.08

**Table 2:** Design parameters used for second scale octahedrons.

Design Parameters	Value
Bars length (mm)	0.7
Bars radius (mm)	0.01
Number of octahedrons	9

**Table 3:** Process parameters used to determinate the optimized ones.

**Laser Power (W)**	150	200	250	300	350	400
**Exposure Time (µs)**	20	35	50	65	80	95

**Table 4:** Optimized process parameters used for the L-PBF process.

Process Parameters	Value
Laser power (W)	150
Exposure time (µs)	55

**Table 5:** Geometries properties.

Properties	Geometry a	Geometry b	Geometry c	Geometry d
Volume (mm^3^)	4.4 × 10^−4^	5.278 × 10^−3^	7.1 × 10^−3^	4.2
Mass (g)	3.6 × 10^−7^	4.3 × 10^−3^	5.8 × 10^−3^	3.4 × 10^−4^
Directional deformation (*Z* axis) (mm)	−7.51 × 10^−3^	−9.02 × 10^−7^	−2.5 × 10^−3^	−2.32 × 10^−3^
Minimun combined stress (MPa)	−6.5 × 10^5^	−9.06 × 10^3^	−3.75 × 10^3^	−2.21 × 10^5^
Maximun combined stress (MPa)	6.09 × 10^5^	8.7 × 10^3^	1.01 × 10^5^	2.05 × 10^3^
Load/Weight	1.24 × 10^2^	2.36 × 10^−2^	1.75 × 10^−2^	1.24 × 10^2^
Relation (Gpa/kg) = Young Module/Weigth	2.56 × 10^5^	4.86 × 10^1^	3.61 × 10^1^	2.56 × 10^5^
